# PubMLST for Antigen Allele Mining to Inform Development of Gonorrhea Protein-Based Vaccines

**DOI:** 10.3389/fmicb.2018.02971

**Published:** 2018-12-07

**Authors:** Benjamin I. Baarda, Ryszard A. Zielke, Robert A. Nicholas, Aleksandra E. Sikora

**Affiliations:** ^1^Department of Pharmaceutical Sciences, College of Pharmacy, Oregon State University, Corvallis, OR, United States; ^2^Department of Pharmacology, The University of North Carolina at Chapel Hill, Chapel Hill, NC, United States; ^3^Department of Microbiology and Immunology, The University of North Carolina at Chapel Hill, Chapel Hill, NC, United States; ^4^Vaccine and Gene Therapy Institute, Oregon Health and Science University, Beaverton, OR, United States

**Keywords:** *Neisseria gonorrhoeae*, vaccine, PubMLST, phylogenetic analysis, structural mapping, crystal structure, MtrE, BamA

## Abstract

*Neisseria gonorrhoeae* (*Ng*) is a human-specific pathogen and the etiological agent of gonorrhea, a sexually transmitted infection with a significant global health burden. While often asymptomatic, untreated gonorrhea can lead to pelvic inflammatory disease, ectopic pregnancy, infertility, and increased transmission/acquisition of HIV. A protective gonorrhea vaccine may be the only way to control disease transmission in the future due to the inexorable development of antibiotic resistance. Subunit antigens are proven candidates for vaccine development due to their safety, cost-effectiveness, and rapid preparation. To inform protein-based gonorrhea vaccine design by including different antigen variants, herein we present bioinformatics mining of alleles and single nucleotide/amino acid polymorphisms using DNA/protein sequences of all *Ng* isolates deposited into the PubMLST database and MtrE and BamA as model antigens. We also present phylogenetic analyses that can be performed using sequence data to gain insights into the evolutionary relationships between the polymorphisms found among the population of isolates using a convenient tool: Molecular Evolutionary Genetics Analysis (MEGA) software. Finally, we perform antigen polymorphism mapping onto the MtrE and BamA structures. This methodology can be applied for rational vaccine design to increase vaccine coverage and cross-protection by heteroligand presentation achieved via inclusion of diverse antigen variants and is relevant to over 100 different species and genera deposited into the PubMLST family of databases.

## Introduction

Gonorrhea is a sexually transmitted infection caused by *Neisseria gonorrhoeae* (*Ng*), a human-specific pathogen that infects over 78 million people worldwide (Newman et al., 2015). Gonorrhea is a debilitating disease with clinical manifestations including cervicitis, urethritis, proctitis, conjunctivitis, or pharyngitis. Women are disproportionally affected by gonorrhea due to the high rate of asymptomatic infections and consequences of infection, which, if left untreated or if improperly treated, can lead to endometritis, pelvic inflammatory disease, pregnancy complications, and infertility. Antibiotics have been instrumental in treating infections in the past eight decades, but *Ng* has gradually acquired resistance to all antimicrobials used in the clinic (Alirol et al., 2017). The currently recommended treatment is dual therapy with ceftriaxone and azithromycin, but *Ng* resistant to each of these antibiotics continue to arise worldwide (World Health Organization [WHO], 2011, 2012, 2015; Centers for Disease Control and Prevention [CDC], 2013, 2015; Newman et al., 2015; Alirol et al., 2017), including recently identified isolates that are resistant to both antibiotics (Fifer et al., 2016; Public Health England, 2018).

While options for developing effective antimicrobials are increasingly limited, an effective gonorrhea vaccine could significantly reduce infection burden and control gonorrhea globally, a goal that has been advocated by public health organizations and researchers (Zhu et al., 2011; ECDC, 2012; Jerse and Deal, 2013; Jerse et al., 2014; WHO, 2016; Zielke et al., 2016; CDC, 2017; Rice et al., 2017; Unemo and Sikora, 2017). Unsuccessful early attempts at using killed cells or purified pilin as vaccine candidates (Zhu et al., 2011), together with the realization that *Ng* readily undergoes phase and antigenic variation for many surface antigens and employs sophisticated immune evasion mechanisms, put vaccine research on hold for several decades. However, discovery of promising proteome-derived vaccine candidates (Zielke et al., 2014, 2016, 2018; Sikora et al., 2018), development of small animal models for systematic testing of vaccine formulations, and the recent success of the MenZB and 4CMenB vaccines for Group B meningitis, the major group of *N. meningitidis* (*Nm*) lacking a capsule-based vaccine (Petousis-Harris et al., 2017), indicates that developing an effective gonorrhea vaccine is within reach. Retrospective data suggested that MenZB had a 31% effectiveness against gonorrhea in the immunized cohort (Petousis-Harris et al., 2017). In support of this finding, immunization of mice with the similar 4CMenB vaccine significantly increased *Ng* clearance in the mouse model of gonorrhea (*P* < 0.0001), and sera from 4CMenB-vaccinated mice cross-reacted with several proteins of the *Ng* outer membrane, including MtrE, BamA, Opa, and PilQ, suggesting that these are the cross-protective proteins (Connolly et al., 2018).

Subunit antigens are attractive candidates for vaccine development due to their safety, cost-effectiveness, and rapid preparation (Bae et al., 2009; Fischer et al., 2010). To inform protein-based gonorrhea vaccine design by including the most prevalent alleles of the target antigens to provide broad protection, herein we present bioinformatics mining of all *Ng* isolates deposited into the PubMLST database^1^ to identify alleles and single nucleotide/amino acid polymorphisms of the model antigens MtrE and BamA. PubMLST is a curated database that hosts multi-locus sequence typing (MLST) data, isolate information, and progressively increasing numbers of whole genome sequences for many microbes including *Ng* and *Nm* (Maiden et al., 1998; Jolley et al., 2004; Jolley and Maiden, 2010). Additionally, allelic variation data are available for over 2500 loci, including the core *Neisseria* genome (Jolley et al., 2018). One limitation of the database is that gene and genome sequences are deposited from specific studies that may have inherent sampling biases. As a result, the frequency of a particular variant in the database is not necessarily representative of the variant’s global distribution. Nevertheless, the PubMLST database is the largest collection of *Neisseria* genetic and genomic information available and can therefore be instrumental in enabling rational vaccine design by identifying allelic variation of protein antigens in order to formulate vaccines with the broadest coverage.

The model vaccine antigens used in our study, MtrE and BamA, are integral β-barrel outer membrane proteins (OMPs) with excellent properties for subunit vaccine candidates for *Ng*: (i) they both have surface-exposed loops that are highly conserved, (ii) the proteins are not phase or sequence variable, (iii) the proteins are expressed in all *Ng* isolates examined to date, (iv) they elicit broad cross-reactive bactericidal antibodies, and (v) MtrE and BamA play pivotal roles in bacterial pathogenesis and physiology, respectively (Delahay et al., 1997; Lee and Shafer, 1999; Veal et al., 2002; Jerse et al., 2003; Rouquette-Loughlin et al., 2005; Zhu et al., 2011; Lei et al., 2014; Zielke et al., 2014, 2016; Bakelar et al., 2016; Rice et al., 2017). MtrE is the outer membrane channel for three *Ng* efflux pumps: MtrCDE, the major antibiotic efflux pump; FarAB, which exports fatty acids; and MacAB, which exports macrolides (Delahay et al., 1997; Lee and Shafer, 1999; Veal et al., 2002; Rouquette-Loughlin et al., 2005; Lei et al., 2014). MtrE is intimately involved in antibiotic resistance; e.g., knockout of MtrE in penicillin- or ceftriaxone-resistant strains markedly decreased the MIC of penicillin and ceftriaxone (Veal et al., 2002; Golparian et al., 2014), indicating that the pump is crucial in resisting the effects of antibiotics. Furthermore, MtrE is important for resistance to innate effectors, including antimicrobial peptides, fatty acids, bile salts, progesterone, and long-chain fecal lipids (Unemo and Shafer, 2014), and crucial for experimental infection in the mouse model of gonorrhea (Jerse et al., 2003). BamA is the central component of the β-barrel assembly machinery (BAM) responsible for the biogenesis of β-barrel OMPs, including BamA itself and MtrE (Knowles et al., 2009; O’Neil et al., 2015; Bakelar et al., 2016; Noinaj et al., 2017). In contrast to the *Escherichia coli* BAM protein complex, BAM in *Ng* contains the cell-surface displayed lipoprotein BamE, the accessory RmpM protein, and no BamB ortholog (Volokhina et al., 2009; Zielke et al., 2016; Sikora et al., 2018). BamA is essential for *Ng* cell viability (Zielke et al., 2016).

In this study, we present methodology for antigen allele mining that can be applied to rational vaccine design to increase vaccine coverage and cross-protection by inclusion of the most prevalent antigen alleles, particularly at extracellular regions exposed on the surface of the bacteria.

## Methods

### Use of PubMLST *Neisseria* Database for Allele Mining

#### Locus I dentification

**FIGURE 1:**
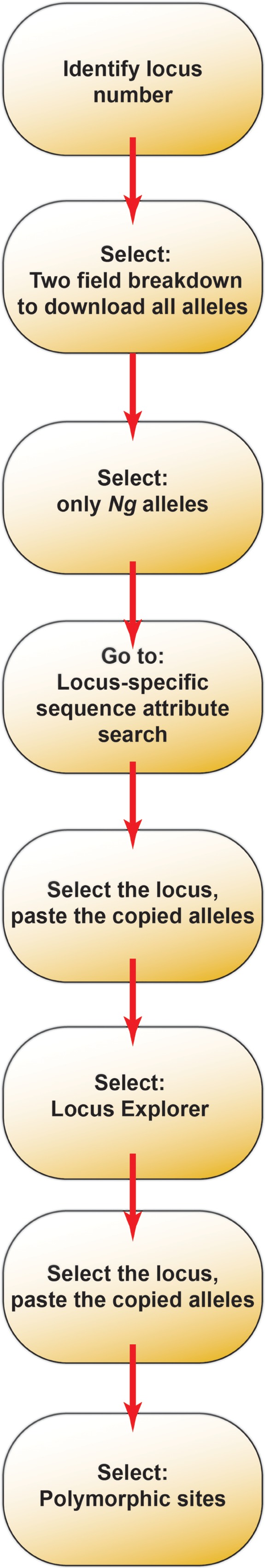
A schematic of the allele mining workflow using the *Neisseria* PubMLST database as a resource.

A schematic of the allele mining workflow can be found in Figure 1, with procedural details in the following sections. It should be noted that the website interface may change at some point after publication of this article. However, an up-to-date user’s manual can be found at http://bigsdb.readthedocs.org/, which should be referenced if difficulties arise. The steps provided here can serve as a guideline when using the manual and can identify the broader topics under which more specific information can be found. From the *Neisseria* PubMLST homepage^1^ (Supplementary Figure S1A), determine the locus identifier for each antigen by navigating to the “Sequence and profile definitions” link, then clicking on the “Sequence query” link to search the database. Select “All loci” from the drop-down menu, and order results by locus (default parameters; Supplementary Figure S1B). Copy and paste the nucleotide sequence for each antigen into the query sequence box, and submit the query. If exact matches for the sequence are found, the results page will identify the locus and the allele that matches the query sequence. If exact matches are not present in the database, the closest alignments will be returned. In the example used in Supplementary Figure S1 (*N. gonorrhoeae* MtrE using the FA1090 *mtrE* gene sequence to query the database), two exact matches were returned: “NEIS1632 (mtrE),” which represents the nucleotide sequence, and “NEISp1632,” which represents the amino acid sequence of the translated protein (Supplementary Figure S1C). Translated protein loci are available for only a subset of loci in the database, but are available for both antigens investigated in the use case presented here. All following steps were performed with the translated protein locus, but the steps are identical for examining nucleic acid-only loci, with the exception that nucleic acid sequences should be translated prior to allele mapping and phylogenetic analyses.

#### Selection of Species-Specific Alleles

A two-field breakdown can be used to identify all alleles present in *Ng*, rather than in all *Neisseria* species existing in the database. From the homepage, select the “Isolates” page, and navigate to the “Two field” link under the Breakdown section of the Isolates page (Supplementary Figure S2A, red box). To perform the two-field breakdown of the dataset, list all isolates (Supplementary Figure S2B, red arrow), set Field 1 to the locus identifier (NEISp1632 in the use case), and set Field 2 to “species.” All other options should be maintained at their default settings. After submission, a table listing each allele for each species in the database, as well as the number of isolates associated with the alleles, will be generated. At the bottom of the page, select “Excel format” to download the table as an Excel spreadsheet (Supplementary Figure S2C, orange box). In the spreadsheet, select the row with species names, click “Sort & Filter” in the Excel toolbar (Supplementary Figure S2D, blue arrow), and select “Filter” to enable sorting by species. Click on the filter arrow in the right corner of the filtered cell of the species of interest and select “Sort Descending” (Supplementary Figure S2D, orange arrow) to acquire a list of all alleles found for the selected species, sorted from highest prevalence to lowest. Note that in the results table for both MtrE and BamA, allele 0 is returned, which is associated with 23 and 5 isolates, respectively. Allele 0 represents a null value where the specific sequence was not discovered during automated scanning of the genome; therefore, we have treated this allele as though it is included with the isolates with “No value” for the locus and removed the isolates associated with it from the prevalence calculations in the use case below. BamA is absolutely required for gonococcal viability (Zielke et al., 2016). While MtrE is not essential (Delahay et al., 1997), MtrE-deficient mutants are significantly attenuated *in vivo* (Jerse et al., 2003). If the 23 isolates with a null value for the MtrE locus are in fact lacking MtrE, they are unlikely to contribute substantially to a productive infection. In light of these lines of evidence, isolates with a null value for either protein are likely to be mis-annotated. The analysis described here provides information on the overall conservation of the antigen, based on the number of alleles, as well as each allele’s distribution within the population of isolates present in the database.

#### Species-Specific Polymorphism Analysis

From the PubMLST *Neisseria* database homepage, navigate to the “Sequence and profile definitions” page and click the “Locus-specific sequence attribute search” link. Select the locus of interest from the drop-down menu (Supplementary Figure S3A). This action will reload the page; data should therefore not be entered prior to locus selection. To perform an analysis of the alleles of interest, click the “Modify form options” box in the top right corner of the form page (Supplementary Figure S3A, red box) and click the plus sign next to the “Allele id list box” option (Supplementary Figure S3B, red arrow). This preference can be saved by clicking the save icon in the bottom left corner of the option menu so that the option will not need to be selected every time the analysis is performed. From the Excel spreadsheet generated in the previous section, copy the list of alleles for which the analysis is desired, ignoring the “No value” entry. Paste the allele list into the “Allele id list” box on the locus analysis form (Supplementary Figure S3C) and click submit. A table of results will be generated that lists information on each allele, including any comments entered for a particular allele (Supplementary Figure S3D). From this point, the sequences (nucleotide or amino acid) can be exported in FASTA format for alignment and phylogenetic analysis, as described below, or the table can be exported to Excel or in a tab-delimited format.

To view polymorphism data in a graphical format, click the “Locus Explorer” button (Supplementary Figure S3D, **bottom**). On the subsequent page, the correct alleles will automatically be selected. **DO NOT** click the “All” button, as that will select all alleles for the locus, not just the alleles for the species of interest (Supplementary Figure S4A). After submission, a graphic of polymorphism data will be generated, as well as a table with amino acid or nucleotide frequencies at each position of the sequence. A portion of this table is shown in Supplementary Figure S4B. If the results are not automatically displayed, click the “Locus schematic (HTML format)” link to navigate to the results page. Not only does the polymorphism analysis provide data on the level of conservation of each antigen under consideration, but the information gathered from the table of nucleotide/amino acid frequencies can also be employed, as in the use case below, to map polymorphism data to protein structure where available, thus facilitating structure-based vaccinology.

#### Phylogenetic Analysis of Antigen Variants

Phylogenetic analyses can be performed using the sequence data to gain insights into the evolutionary relationships between the polymorphisms found among the population of isolates. A convenient tool for these analyses is the Molecular Evolutionary Genetics Analysis (MEGA) software version 7, available as a free download from https://www.megasoftware.net (Kumar et al., 2016). MEGA can be downloaded in either a command-line format or a graphical user interface, the latter of which will be used in this report. From the previous section, after submitting the list of alleles on the locus attribute form, click the “Export: FASTA” box (Supplementary Figure S3D, above “Locus Explorer” button), then click the “Download” link on the subsequent page. All allele sequences will be returned in FASTA format. In MEGA, click the “Align” button in the main application window, click Edit/Build Alignment (Supplementary Figure S5A), select the “Create a new alignment” option on the pop-up window, click “OK,” and select whether the alignment will use DNA or protein sequences.

To build the alignment, select and copy all of the allele sequences generated in FASTA format, and paste them into the MEGA Alignment Explorer window (if using keyboard shortcuts, Mac users must use ctrl+v to paste, rather than the typical command+v keystrokes). The window will be populated with the sequences of interest, including the locus identifier and the allele number in the “Species/Abbrv” field (Supplementary Figure S5B). If the sequences are in the nucleic acid format, a tab will be present above the sequence data labeled “Translated Protein Sequences.” Click this tab to generate protein sequence data for phylogenetic analyses, then select “Yes” to use the standard genetic code for translation. When translation is complete, the sequences can be aligned with either the ClustalW or the MUSCLE algorithms either by selecting the desired algorithm from the Alignment menu on the menu bar, or by clicking one of the icons that look like a “W” or an arm in the toolbar (Supplementary Figure S5B, Red box). In the use case presented in this report, we have aligned the sequences using ClustalW. When the desired algorithm has been chosen, use the subsequent dialog box to select all sequences (this dialog box will appear only if no sequences have been selected for alignment; all sequences are selected by default after pasting). MEGA will next provide the option of changing the alignment parameters (Supplementary Figure S5C). We have maintained the default settings in the use case presented here. To align the sequences, select “OK.” The software will generate an initial pairwise alignment and will then perform a multiple alignment. After the process is complete, the software will use asterisks to designate which residues are conserved in all sequences (Supplementary Figure S6A, row of gray boxes immediately above alignment data), and the display preferences can be altered to highlight residues conserved in 50, 60, 70, 80, 90, or 100% of sequences (click “Display” on the menu bar, then “Toggle Conserved Sites” and select the desired level of conservation).

To perform phylogenetic analyses, click “Data” in the menu bar, and select the “Phylogenetic Analysis” option (Supplementary Figure S6A). The software will process the data to enable the analysis. In the main window, click “Phylogeny” in the toolbar, select “Construct/Test Maximum Likelihood Tree” (Supplementary Figure S6B), and click “Yes” in the subsequent dialog box to use the currently active data. MEGA will again provide the option to change parameters. We have kept the default settings for this analysis as well, with the exception that we selected to test the phylogenies with 500 Bootstrap replicates (Supplementary Figure S6C). After “Compute” is selected, the software will generate the maximum likelihood tree, which can then be exported as an enhanced metafile image for use in Adobe Illustrator or in other formats for manipulation in tree-editing software, which is not discussed here.

#### Antigen Polymorphism Structural Mapping

**FIGURE 2:**
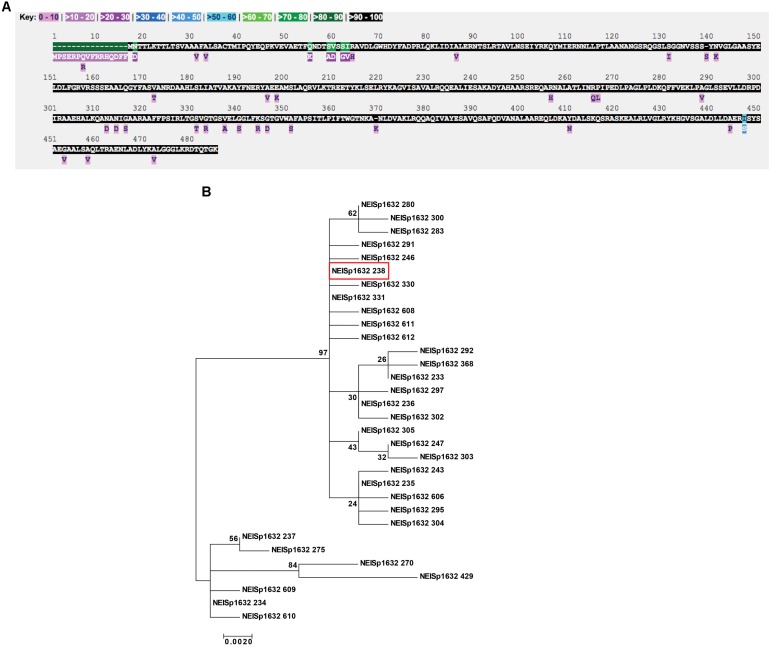
Polymorphism and phylogenetic analyses of *N. gonorrhoeae* MtrE. **(A)** Inspection of MtrE amino acid polymorphisms across 3,908 *N. gonorrhoeae* isolates for which data exists for the NEISp1632 locus revealed the presence of 32 variants with 53 polymorphic sites across the 485 positions in the polymorphism analysis. **(B)** Phylogenetic analysis of the amino acid sequences revealed the two clusters of variants with the main cluster comprised of 25 variants, including the most common gonococcal variant, 238, shown in red box.

Using the polymorphism data generated after clicking on “Locus Explorer,” the polymorphisms (which are color-coded as shown in Figure 2) can be mapped to the crystal structure of the protein provided that the structure is known. The following description is for MtrE, and uses PyMol ^2^ to visualize the structure. The coordinates for MtrE (4mt0; Lei et al., 2014), which can be downloaded from PubMed, are opened in PyMol. Under “H” in the right panel, select “Hide Everything,” then under “S,” select “Show as cartoon.” The color of the entire structure can be chosen under “C” (in the use case below the structure was colored as “gray50”). To mark polymorphic residues, display the sequence (Display > Sequence On), select all of the amino acids from one group (e.g., 0–10%), rename selection (i.e., 0–10), and color these according to your color scheme (in the use case below the color violet was used for this group). Repeat for the other groupings. To display spheres, right-click on each of the colored residues in the structure and select atom > show > spheres (one can also do this in the command line with “show spheres, /4mt0//A/res1+res2+…/CA,” where res is the number of the residue). Finally, to obtain the trimer, under “A,” select generate > show symmetry mates > within 4 A. Remove all of the subunits except for those that complete the trimer by selecting “delete object” under “A.” To generate a high resolution.png file, use the command “ray 2400, 2400;png filename.png” and a .png file is created on your home page. The background can be changed to white under Display > Background > White, and can be removed by toggling off “Opaque Background.” The .png file can be imported into Adobe Illustrator for creation of figures.

## Use Case

In this section, we have used the methods described above to perform each of the analyses on two promising, broadly conserved vaccine candidates, MtrE and BamA.

### Phylogenetic Analysis and Structure Mapping of MtrE Polymorphisms

Examination of MtrE amino acid polymorphisms across 3,908 *N. gonorrhoeae* isolates for which data exists for the NEISp1632 locus revealed the presence of 32 variants with 53 polymorphic sites across the 485 positions in the polymorphism analysis. Large portions of the protein were conserved among all isolates in the database (Figure 2A). The most common variant was 238, which represents 30% of all *N. gonorrhoeae* MtrE sequences. Protein variant 238 and four others account for 98% of the diversity of gonococcal MtrE: variant 233, 21%; variant 235, 21%; variant 236, 13%; and variant 234, 13% (NEISp1632 two-field breakdown; Supplementary Table S1).

Phylogenetic analysis of the amino acid sequences revealed the existence of two clusters of antigen variants (Figure 2B). The main cluster was composed of 25 variants that appeared to be closely related, including the most common gonococcal variant, 238 (Figure 2B, red box). The secondary cluster was made up of six variants that contain a 16 amino acid insertion at the beginning of the protein sequence (variants 234, 237, 270, 429, 609, and 610) and variant 275, which does not possess the insertion but nonetheless contains portions of its sequence with similarities to the other six variants in the secondary cluster. It is possible that this “insertion” is actually a mis-annotation that includes sequence upstream of the translation start site, especially considering that all of the longer sequences also contain a methionine residue at the start site annotated for the other sequences. Automated annotation may have detected an alternate start site in the affected sequences that has not been manually curated. While it is possible that these variants represent true MtrE sequence diversity among the population of *N. gonorrhoeae* isolates present in the database, the result of this extension would be to increase the length of the signal sequence, which is removed during transport of the protein across the cytoplasmic membrane by the secretory machinery and thus not present in the mature protein.

Similar analyses performed on MtrE sequences from all *Neisseria* isolates in the database revealed the presence of 492 protein variants with 298 polymorphic sites. As observed for MtrE in *N. gonorrhoeae*, the majority of the sequence was conserved in 90–100% of variants, with numerous low-prevalence polymorphisms (Supplementary Figure S7). A phylogenetic tree constructed from all MtrE sequences again indicated that MtrE variants are closely related among *Neisseria* with the exception of variant 75, which originated from an isolate of *N. animalis* and formed an outgroup in the analysis (Supplementary Figure S8). Variant 2, exclusively associated with *N. meningitidis* isolates, was the most common *Neisseria* MtrE variant (2,467 of 16,363 isolates for which data exists for this locus; marked with a red box in Supplementary Figure S8).

**FIGURE 3:**
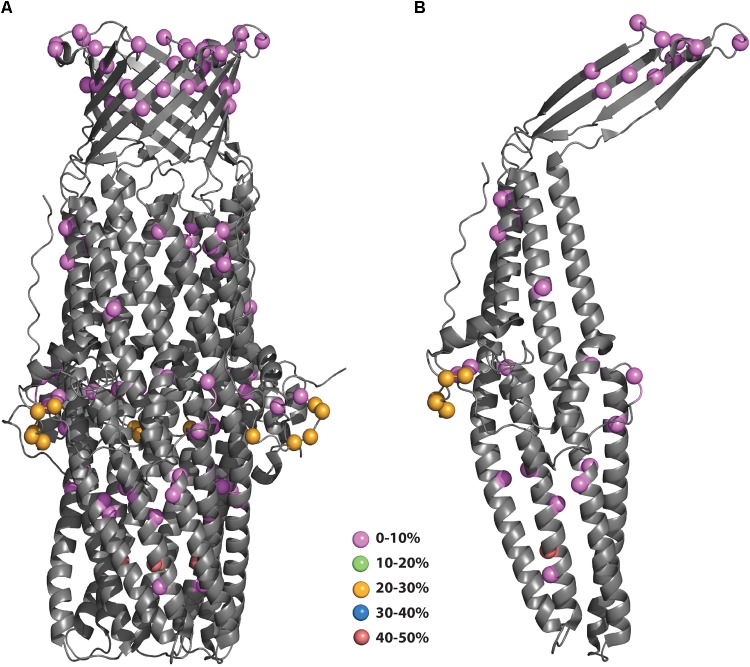
**(A,B)** Polymorphisms of *N. gonorrhoeae* MtrE mapped onto the crystal structure. The polymorphisms from Figure 2A were mapped onto the structure of MtrE (4mt0) and colored according to their prevalence in the population. **(B)** Polymorphisms mapped to MtrE trimer form.

Structure mapping is particularly important for rational vaccine design. The utility of subunit vaccines is that they generate antibodies that would recognize intact bacteria by binding to the surface-exposed regions of the protein. With the goal of increasing vaccine coverage and cross-protection, it is important to select polymorphisms that reside in surface-exposed regions. As shown in Figure 3, the low prevalence polymorphisms are scattered throughout the monomeric MtrE subunit, including 6 residues apparently surface exposed (residues 115, 122, 320, 323, 327, and 329). In contrast, the more prevalent polymorphisms are all located in the periplasm, with all but one clustered on a loop about halfway down the long alpha helical bundles. The most common polymorphism is located on one of the helices near the bottom of the protein (PyMol session files showing the mapping of MtrE polymorphisms to the monomer and trimer forms of MtrE can be found in Supplementary Files S1, S2, respectively).

### Phylogenetic Analysis and Structure Mapping of BamA Polymorphisms

**FIGURE 4:**
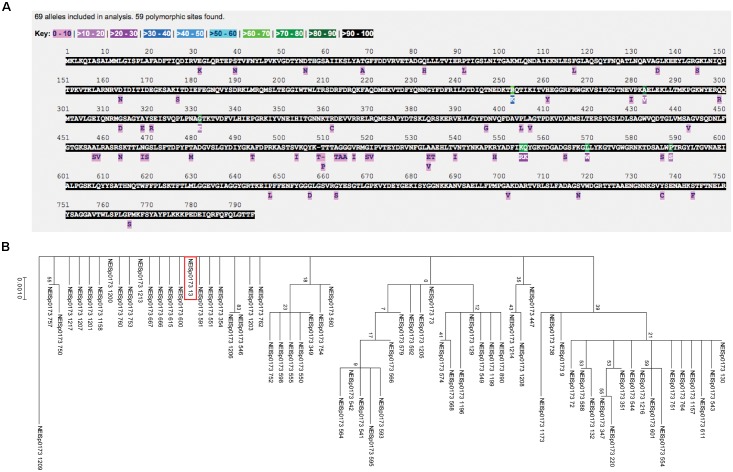
Polymorphism and phylogenetic analyses of *N. gonorrhoeae* BamA. **(A)** Analysis of the NEISp0173 locus revealed 69 variants with 59 amino acid polymorphisms across 3,946 isolates for which sequence data has been deposited. **(B)** Phylogenetic analyses of BamA across *N. gonorrhoeae* showed numerous closely related variants, with several clusters present in the tree. The majority (>94%) of BamA diversity was found in the combination of variant 13 (noted by a red box) with five other variants.

Assessment of the NEISp0173 locus revealed 69 variants with 59 amino acid polymorphisms across 3,946 isolates for which sequence data has been deposited into the database (Figure 4A). Large portions of the BamA amino acid sequence were conserved among 90–100% of protein variants, with low-frequency polymorphisms distributed throughout the sequence. Seven positions were conserved in only 60–80% of variants (green residues in Figure 4A). The most common BamA variant was number 13, which accounted for 67.6% of isolates. Over 94% of BamA diversity was found in the combination of variant 13 with five others: variant 349, 8.0%; variant 73, 7.4%; variant 130, 4.2%; variant 129, 3.7%; and variant 220, 3.3%. Additionally, 55 of the 69 *N. gonorrhoeae* BamA variants were associated with five or fewer isolates (NEISp0173 two-field breakdown; Supplementary Table S2).

An examination of BamA phylogeny across *N. gonorrhoeae* revealed that numerous variants were closely related, with several clusters present in the tree (Figure 4B). The six most common variants (variant 13 is noted by a red box in Figure 4B) were distributed throughout the tree, which could indicate they evolved independently. The most distantly related variant was 1209, which represents two isolates in the database.

Polymorphism analyses of BamA across all *Neisseria* revealed the presence of 744 variants with 622 polymorphic sites across the protein. The amino acid sequence was largely conserved, although numerous low-abundance polymorphisms were present at nearly every position (Supplementary Figure S9). Finally, phylogenetic analysis of all *Neisseria* BamA variants indicated that the majority of BamA variants were closely related. Variant 179, corresponding to an isolate of *N. shayeganii*, formed an outgroup in this analysis. The most common *Neisseria* BamA variant was allele 13, found in 2,666 *N. gonorrhoeae* isolates out of 16,791 *Neisseria* isolates for which BamA sequence data has been deposited (Supplementary Figure S10, identified by a red box). This variant was not present in any other species in the database.

**FIGURE 5:**
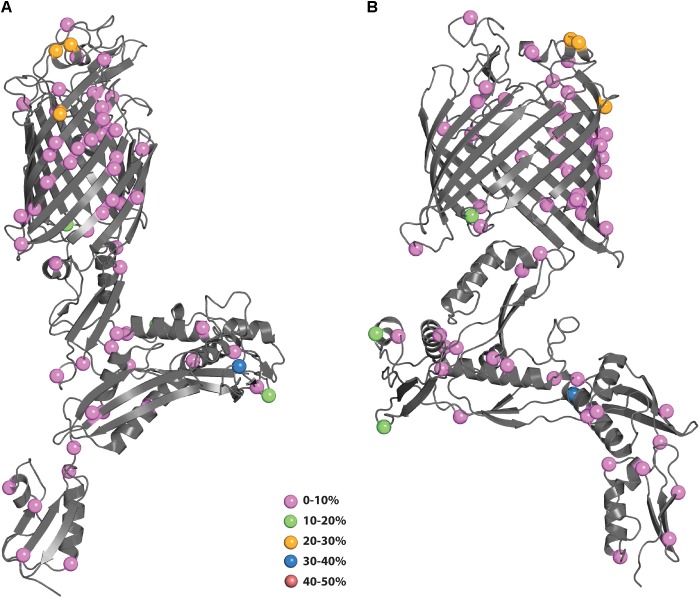
**(A,B)** Polymorphisms of *N. gonorrhoeae* BamA mapped onto the crystal structure. The polymorphisms from Figure 4A were mapped onto the structure of BamA (4k3b) and colored according to their prevalence in the population. **(B)** Shows BamA structure rotated 90° from **(A)**.

Mapping of the polymorphisms onto the *N. gonorrhoeae* BamA structure (4k3b) is shown in Figure 5. The structure of BamA consists of 5 POTRA domains at the *N*-terminus followed by a β-barrel domain at the *C*-terminus (Noinaj et al., 2013). As with MtrE, the polymorphisms are distributed throughout the different domains. There are 6 polymorphisms that map to surface-exposed regions: four of these (residues 547, 563, 735, and 742) are of low prevalence (0–10%), while the other two (residues 553 and 554) are medium prevalence (20–30%). These latter two polymorphisms would be likely candidates to include in a subunit vaccine to increase coverage of strains harboring these existing polymorphisms (PyMol session file showing the mapping of BamA polymorphisms to the structure of BamA can be found in Supplementary File S3).

## Author Contributions

AS, RN, BB, and RZ developed and designed the study, analyzed data, and wrote the manuscript. BB, RZ, and RN performed the analyses. AS and RN designed and executed the figures. BB prepared the supplementary figures.

## Conflict of Interest Statement

The authors declare that the research was conducted in the absence of any commercial or financial relationships that could be construed as a potential conflict of interest.

## Footnotes

1 https://pubmlst.org/neisseria/

2 pymol.org
