# Supplementary material for: PubMLST for Antigen Allele Mining to Inform Development of Gonorrhea Protein-Based Vaccines
Source: Front Microbiol. 2018 Dec 7;9:2971. doi: 10.3389/fmicb.2018.02971 (PMC6292995; doi:10.3389/fmicb.2018.02971)

Supplementary Material

PubMLST for antigen allele mining to inform development of gonorrhea protein-based vaccines

Benjamin I. Baarda,[1#] Ryszard A. Zielke,[1#] Robert A. Nicholas,[2] and Aleksandra E. Sikora[1, 3*]

[1]Department of Pharmaceutical Sciences, College of Pharmacy, Oregon State University, Corvallis, OR, United States.
[2]Departments of Pharmacology and Microbiology and Immunology, University of North Carolina at Chapel Hill, Chapel Hill, NC, USA
[3]Vaccine and Gene Therapy Institute, Oregon Health and Science University, Beaverton, OR, United States.

[#]Authors contributed equally

*** Correspondence:**
Aleksandra E. Sikora
Aleksandra.Sikora@oregonstate.edu
Keywords: *Neisseria gonorrhoeae*, vaccine, PubMLST, phylogenetic analysis, structural mapping, crystal structure, MtrE, BamA

Supplemental Files
Supplemental Table S1. Two-Field Breakdown of MtrE alleles.
Supplemental Table S2. Two-Field Breakdown of BamA alleles.
Supplemental File S1. PyMol session file showing the mapping of MtrE polymorphisms to the structure of the MtrE monomer.
Supplemental File S2. PyMol session file showing the mapping of MtrE polymorphism to the structure of the MtrE trimer.
Supplemental File S3. PyMol session file showing the mapping of BamA polymorphisms to the structure of BamA.

Supplemental Figure S1. Querying the PubMLST database for locus identification. (A) Homepage of the PubMLST website, showing database navigation options. (B) Sequence query form for locus identification (C) Result screen showing alleles that are exact matches for the query sequence.
Supplemental Figure S2. Identification of species-specific alleles. (A) Screenshot identifying link to perform two-field analysis of locus (red box). (B) Options for performing two-field breakdown of locus. Button for selecting all isolates in the database is noted by a red arrow. (C) Table of results for two-field breakdown of locus. Link to export results table to Microsoft Excel is identified by an orange box. (D) Excel sheet of results table. “Sort & Filter” toolbar option to enable filtering to sort the alleles for each species is identified with a blue arrow, and the “Descending” option in the filtered cell is denoted by an orange arrow.
Supplemental Figure S3. Species-specific analysis of locus. (A) Form for selecting alleles of interest for locus under investigation. Menu to modify form options is identified in a red box. (B) View of opened “Modify form options” menu. Select “Allele id list box” option (noted by a red

arrow) to be able to paste a list of alleles for analysis. (C) Form including allele id list box, populated with alleles of interest copied from sorted Excel sheet from Fig. S2D. (D) Results page for alleles of interest. Options to export results in FASTA format or table format can be seen, as well as option to perform polymorphism analysis by selecting “Locus Explorer” button.

Supplemental Figure S4. Polymorphism analysis of alleles of interest. (A) Form to select which alleles to analyze with the Locus Explorer. Note that alleles of interest from previous step (Fig. S3D) are selected by default. (B) Table of polymorphism data indicating number and percentage of alleles with particular amino acids at each position.

Supplemental Figure S5. Alignment of variant sequences to enable phylogenetic analysis using MEGA. (A) Main MEGA window showing option to build an alignment of sequences. (B) Alignment explorer window with sequences downloaded in FASTA format (from Fig. S3D) entered. ClustalW (W icon) and Muscle (flexed arm icon) alignment options are identified in a red box. (C) Window for selecting alignment parameters. Default ClustalW options are shown.

Supplemental Figure S6. Construction of phylogenetic tree. (A) Screenshot showing menu option to enable phylogenetic analysis from aligned sequence data. (B) Toolbar option to construct and test a maximum likelihood tree from the alignment, after enabling phylogenetic analyses. (C) Window for selecting analysis parameters. Default options are shown, except that the Bootstrap method was selected to test the phylogenies, and 500 Bootstrapping iterations were selected.

Supplemental Figure S7. Polymorphism analyses of MtrE across all *Neisseria.* MtrE sequences from all *Neisseria* isolates present in the database represent 492 variants with 298 polymorphic sites.

Supplemental Figure S8. Phylogenetic analysis of all *Neisseria* MtrE variants. A phylogenetic tree was constructed from all 492 *Neisseria* MtrE variants present in the database using the Jones-Taylor-Thornton method to calculate a distance matrix, then applying Neighbor-Join and BioNJ algorithms to generate an initial tree, which was heuristically searched with the Nearest Neighbor-Interchange method. Phylogenies were tested with 500 bootstrap replications, and the highest log-likelihood tree is presented. The most common MtrE variant (variant 2) is noted with a red box.

Supplemental Figure S9. Polymorphism analyses of BamA across all *Neisseria.* BamA sequences from all *Neisseria* isolates present in the database represent 744 variants with 622 polymorphic sites.

Supplemental Figure S10. Phylogenetic analysis of all *Neisseria* BamA variants. A phylogenetic tree was constructed from all 744 *Neisseria* BamA variants present in the database using the Jones-Taylor-Thornton method to calculate a distance matrix, then applying Neighbor-Join and BioNJ algorithms to generate an initial tree, which was heuristically searched with the Nearest Neighbor-Interchange method. Phylogenies were tested with 500 bootstrap replications, and the highest log-likelihood tree is presented. The most common BamA variant (variant 13) is noted with a red box.

Figure S1

A

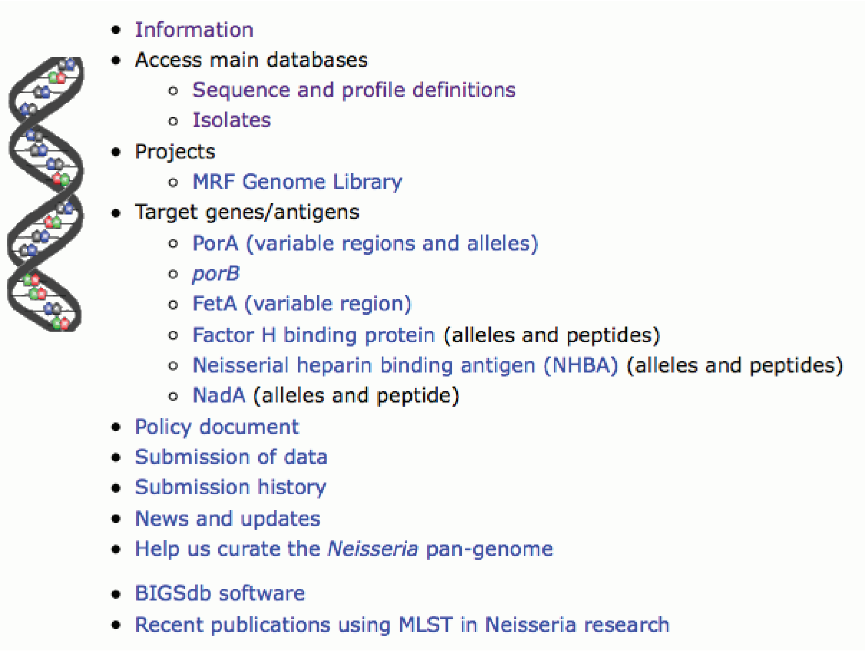

B

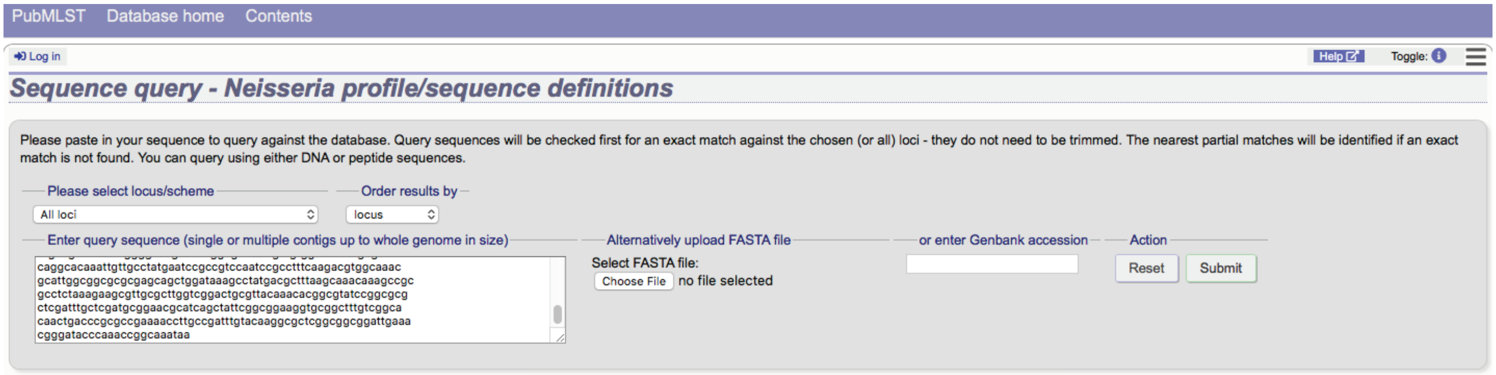

C

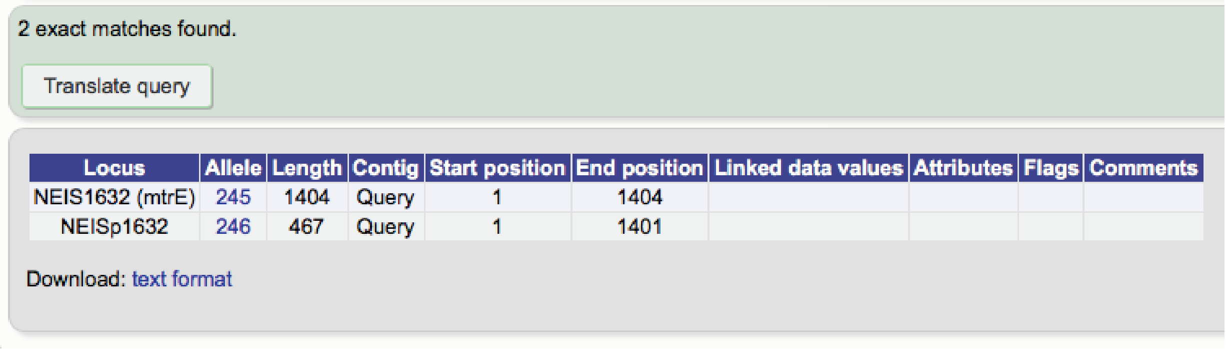

Figure S2

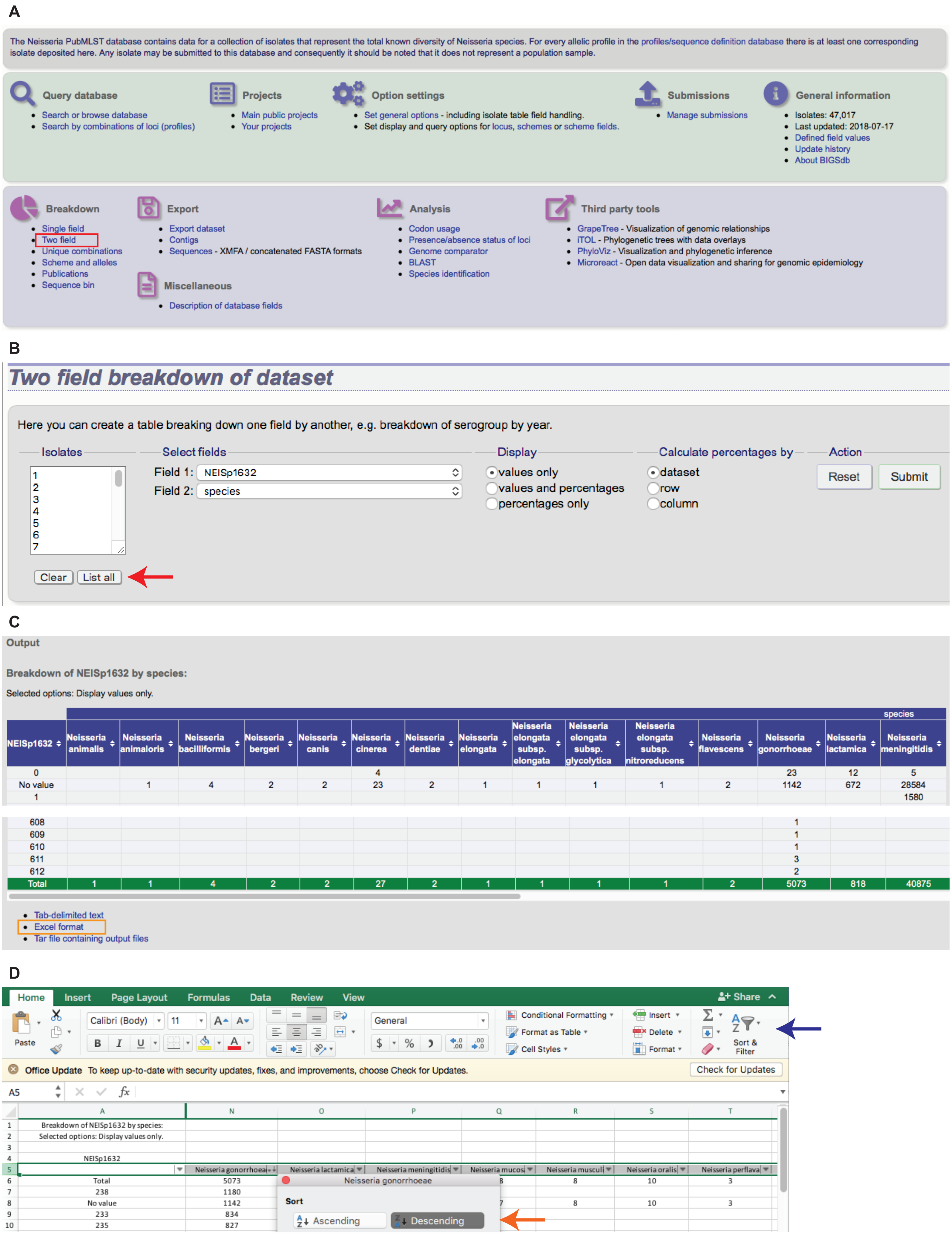
A
The Neisseria PubMLST database contains data for a collection of isolates that represent the total known diversity of Neisseria species. For every allelic profile in the profiles/sequence definition database there is at least one corresponding isolate deposited here. Any isolate may be submitted to this database and consequently it should be noted that it does not represent a population sample.
Query database
Search or browse database
Search by combinations of loci (profiles)
Projects
Main public projects
Your projects
Option settings
Set general options - including isolate table field handling.
Set display and query options for locus, schemes or scheme fields.
Submissions
Manage submissions
General information
Isolates: 47,017
Last updated: 2018-07-17
Defined field values
Update history
About BIGSdb
Breakdown
Single field
Two field
Unique combinations
Scheme and alleles
Publications
Sequence bin
Export
Export dataset
Contigs
Sequences - XMFA / concatenated FASTA formats
Miscellaneous
Description of database fields
Analysis
Codon usage
Presence/absence status of loci
Genome comparator
BLAST
Species identification
Third party tools
GrapeTree - Visualization of genomic relationships
iTOL - Phylogenetic trees with data overlays
PhyloViz - Visualization and phylogenetic inference
Microreact - Open data visualization and sharing for genomic epidemiology
B
Two field breakdown of dataset
Here you can create a table breaking down one field by another, e.g. breakdown of serogroup by year.
Isolates
Select fields
Field 1: NEISp1632
Field 2: species
Display
values only
values and percentages
percentages only
Calculate percentages by
dataset
row
column
Action
Reset
Submit
Clear
List all
C
Output
Breakdown of NEISp1632 by species:
Selected options: Display values only.
species
NEISp1632
Neisseria animalis
Neisseria animaloris
Neisseria bacilliformis
Neisseria bergeri
Neisseria canis
Neisseria cinerea
Neisseria dentiae
Neisseria elongata
Neisseria elongata subsp. elongata
Neisseria elongata subsp. glycolytica
Neisseria elongata subsp. nitroreducens
Neisseria flavescens
Neisseria gonorrhoeae
Neisseria lactamica
Neisseria meningitidis
Total
Tab-delimited text
Excel format
Tar file containing output files
D
Home
Insert
Page Layout
Formulas
Data
Review
View
Share
Paste
Calibri (Body)
General
Conditional Formatting
Format as Table
Cell Styles
Insert
Delete
Format
Sort & Filter
Office Update To keep up-to-date with security updates, fixes, and improvements, choose Check for Updates.
Check for Updates
Breakdown of NEISp1632 by species:
Selected options: Display values only.
NEISp1632
Neisseria gonorrhoeae
Neisseria lactamica
Neisseria meningitidis
Neisseria mucos
Neisseria musculi
Neisseria oralis
Neisseria perflava
Total
No value
Sort
Ascending
Descending

Figure S3

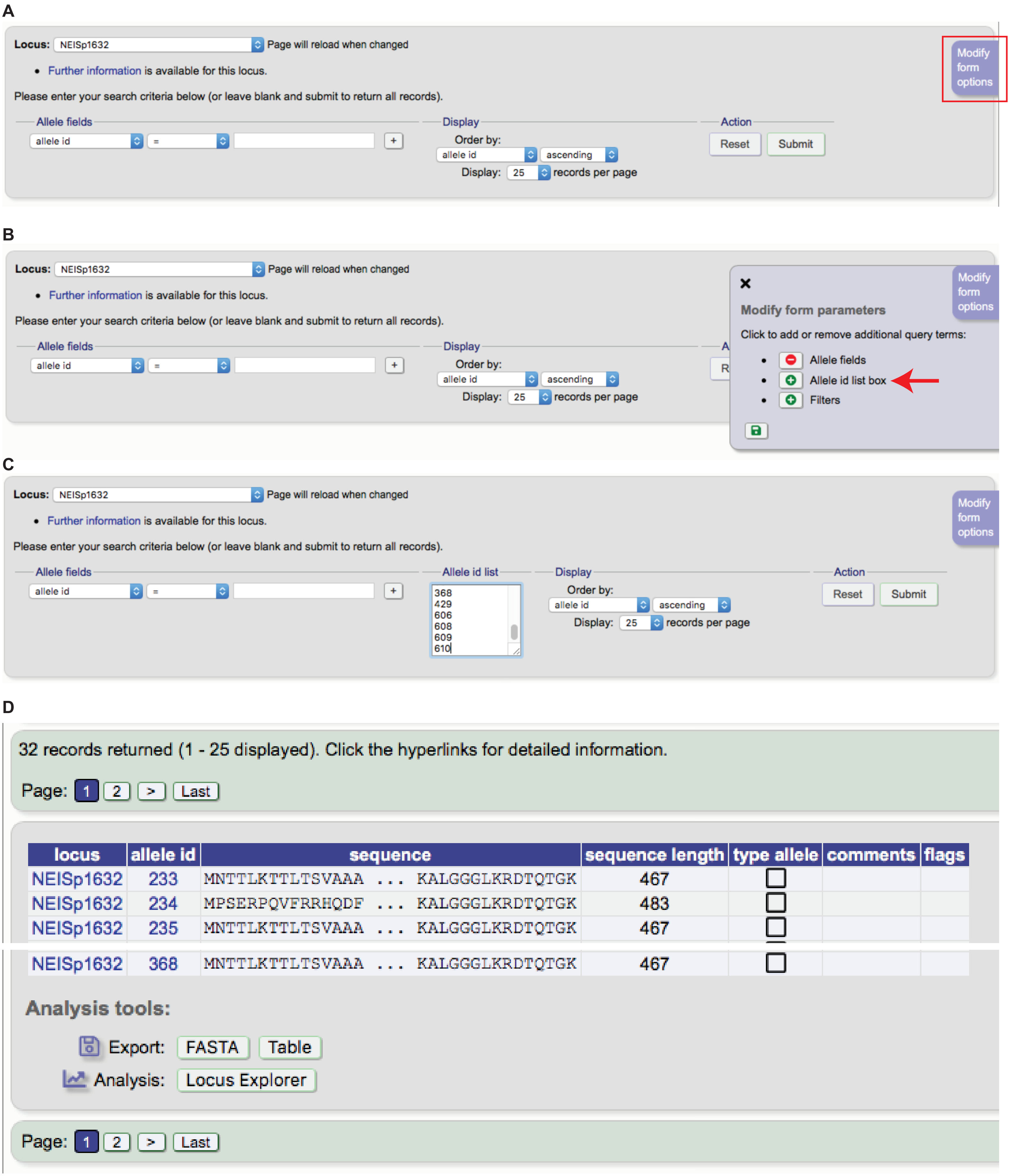

A
Locus: NEISp1632 Page will reload when changed
Further information is available for this locus.
Please enter your search criteria below (or leave blank and submit to return all records).
Allele fields
allele id
=
+
Display
Order by:
allele id
ascending
Display: 25 records per page
Action
Reset
Submit
Modify form options
B
Locus: NEISp1632 Page will reload when changed
Further information is available for this locus.
Please enter your search criteria below (or leave blank and submit to return all records).
Allele fields
allele id
=
Display
Order by:
allele id
ascending
Display: 25 records per page
Modify form parameters
Click to add or remove additional query terms:
Allele fields
Allele id list box
Filters
Modify form options
C
Locus: NEISp1632 Page will reload when changed
Further information is available for this locus.
Please enter your search criteria below (or leave blank and submit to return all records).
Allele fields
allele id
=
Allele id list
368
429
606
608
609
610
Display
Order by:
allele id
ascending
Display: 25 records per page
Action
Reset
Submit
Modify form options
D
32 records returned (1 - 25 displayed). Click the hyperlinks for detailed information.
Page: 1 2 > Last
locus | allele id | sequence | sequence length | type allele | comments | flags
NEISp1632 233 MNTTLKTTLTSVAAA ... KALGGGLKRDTQTGK 467
NEISp1632 234 MPSERPQVFRRHQDF ... KALGGGLKRDTQTGK 483
NEISp1632 235 MNTTLKTTLTSVAAA ... KALGGGLKRDTQTGK 467
NEISp1632 368 MNTTLKTTLTSVAAA ... KALGGGLKRDTQTGK 467
Analysis tools:
Export: FASTA Table
Analysis: Locus Explorer
Page: 1 2 > Last

Figure S4

A

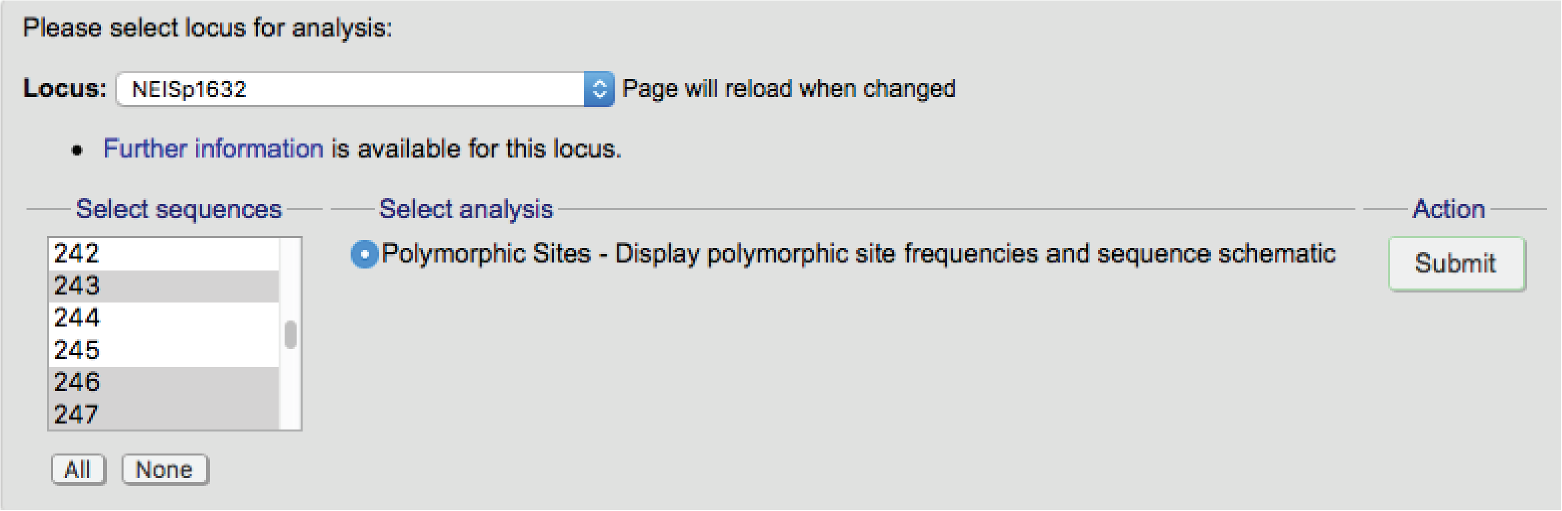

B

Amino acid frequencies

Position	Amino acid																															
	G	A	L	M	F	W	K	Q	E	S	P	V	I	C	Y	H	R	N	D	T	-	%G	%A	%L	%M	%F	%W	%K	%Q	%E	%S	[illegible]
1	0	0	0	6	0	0	0	0	0	0	0	0	0	0	0	0	0	0	0	0	26				18.75							
2	0	0	0	0	0	0	0	0	0	0	6	0	0	0	0	0	0	0	0	0	26											
3	0	0	0	0	0	0	0	0	0	6	0	0	0	0	0	0	0	0	0	0	26										18.75	
4	0	0	0	0	0	0	0	0	6	0	0	0	0	0	0	0	0	0	0	0	26									18.75		
5	0	0	0	0	0	0	0	0	0	0	0	0	0	0	0	0	6	0	0	0	26											
6	0	0	0	0	0	0	0	0	0	0	6	0	0	0	0	0	0	0	0	0	26											
7	0	0	0	0	0	0	0	5	0	0	0	0	0	0	0	0	1	0	0	0	26								15.63			
8	0	0	0	0	0	0	0	0	0	0	0	6	0	0	0	0	0	0	0	0	26											
9	0	0	0	0	6	0	0	0	0	0	0	0	0	0	0	0	0	0	0	0	26					18.75						
10	0	0	0	0	0	0	0	0	0	0	0	0	0	0	0	0	6	0	0	0	26											
11	0	0	0	0	0	0	0	0	0	0	0	0	0	0	0	0	6	0	0	0	26											
12	0	0	0	0	0	0	0	0	0	0	0	0	0	0	0	6	0	0	0	0	26											
13	0	0	0	0	0	0	0	6	0	0	0	0	0	0	0	0	0	0	0	0	26								18.75			
14	0	0	0	0	0	0	0	0	0	0	0	0	0	0	0	0	0	0	6	0	26											
15	0	0	0	0	6	0	0	0	0	0	0	0	0	0	0	0	0	0	0	0	26					18.75						
16	0	0	0	0	6	0	0	0	0	0	0	0	0	0	0	0	0	0	0	0	26					18.75						
18	0	0	0	0	0	0	0	0	0	0	0	0	0	0	0	0	0	25	7	0	0											
31	0	31	0	0	0	0	0	0	0	0	0	1	0	0	0	0	0	0	0	0	0		96.88									
33	0	31	0	0	0	0	0	0	0	0	0	1	0	0	0	0	0	0	0	0	0		96.88									

Figure S5

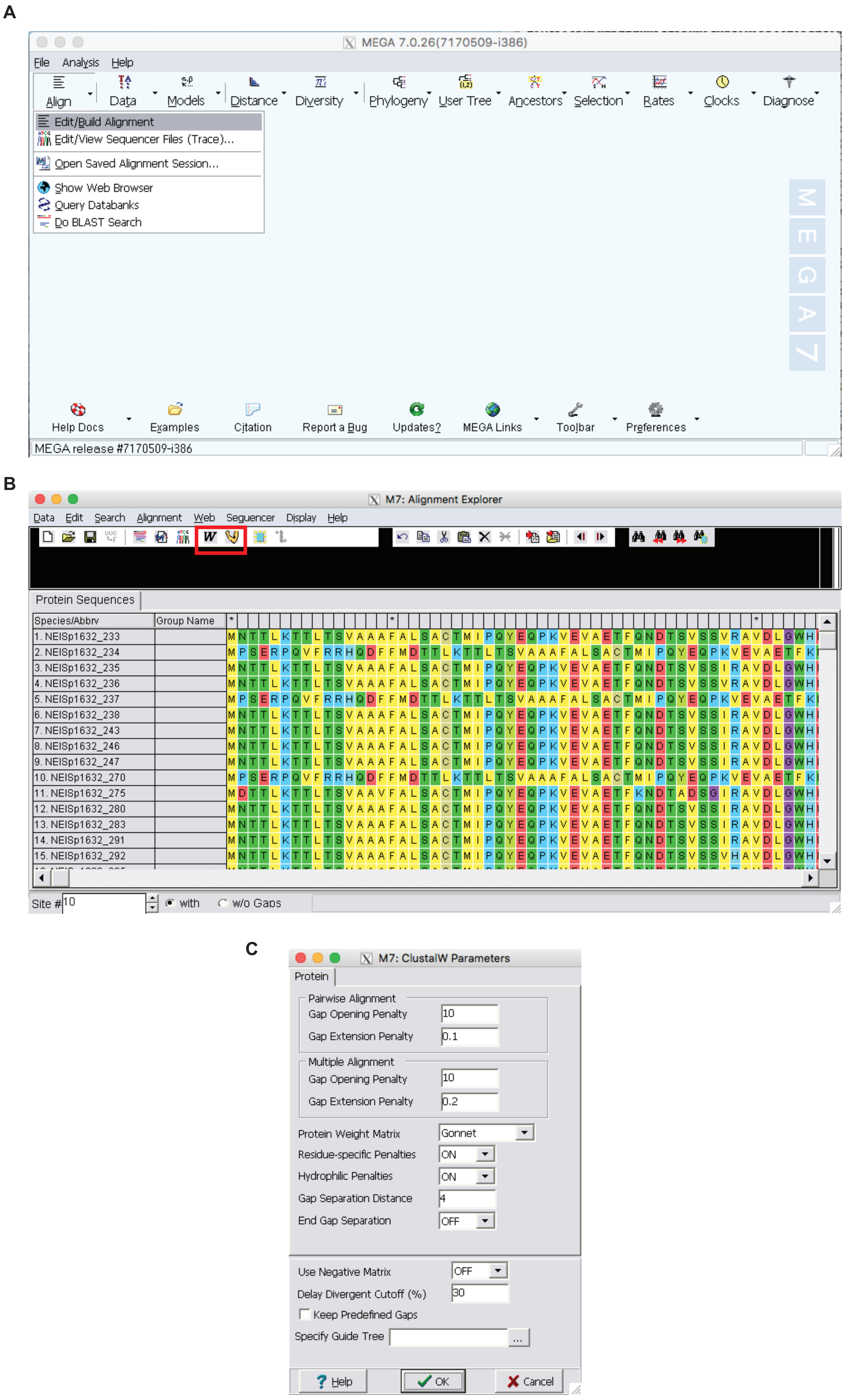
A
MEGA 7.0.26(7170509-i386)
File Analysis Help
Align Data Models Distance Diversity Phylogeny User Tree Ancestors Selection Rates Clocks Diagnose
Edit/Build Alignment
Edit/View Sequencer Files (Trace)...
Open Saved Alignment Session...
Show Web Browser
Query Databanks
Do BLAST Search
MEGA7
Help Docs Examples Citation Report a Bug Updates? MEGA Links Toolbar Preferences
MEGA release #7170509-i386
B
M7: Alignment Explorer
Data Edit Search Alignment Web Sequencer Display Help
Protein Sequences
Species/Abbrv
Group Name
1. NEISp1632_233
2. NEISp1632_234
3. NEISp1632_235
4. NEISp1632_236
5. NEISp1632_237
6. NEISp1632_238
7. NEISp1632_243
8. NEISp1632_246
9. NEISp1632_247
10. NEISp1632_270
11. NEISp1632_275
12. NEISp1632_280
13. NEISp1632_283
14. NEISp1632_291
15. NEISp1632_292
Site # 10
with
w/o Gaps
C
M7: ClustalW Parameters
Protein
Pairwise Alignment
Gap Opening Penalty 10
Gap Extension Penalty 0.1
Multiple Alignment
Gap Opening Penalty 10
Gap Extension Penalty 0.2
Protein Weight Matrix Gonnet
Residue-specific Penalties ON
Hydrophilic Penalties ON
Gap Separation Distance 4
End Gap Separation OFF
Use Negative Matrix OFF
Delay Divergent Cutoff (%) 30
Keep Predefined Gaps
Specify Guide Tree
Help
OK
Cancel

Figure S6

A

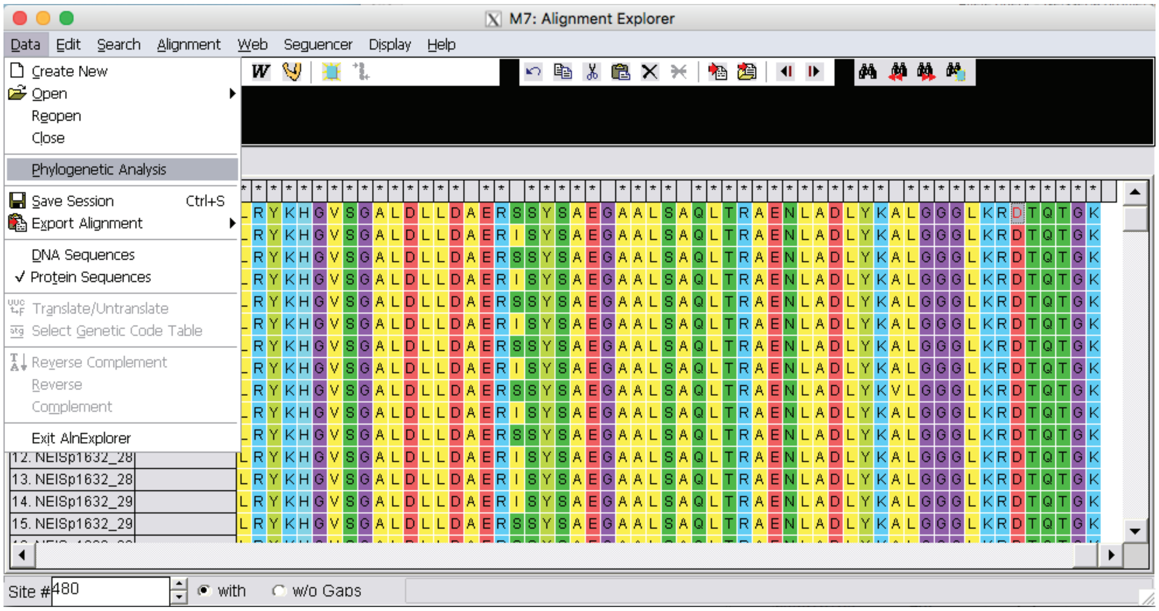

B

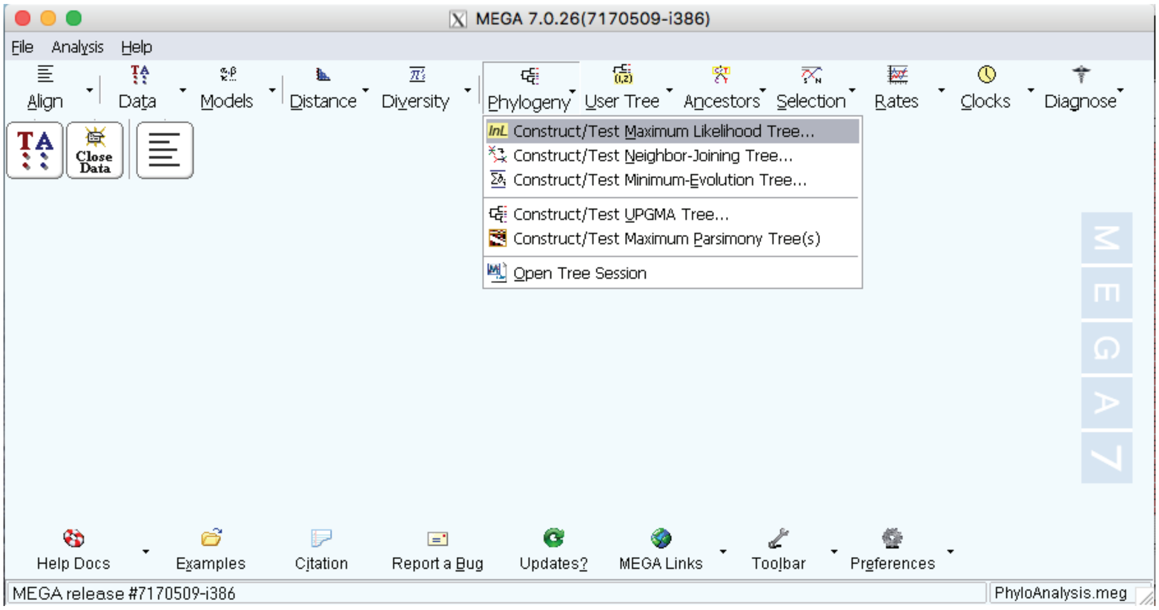

C

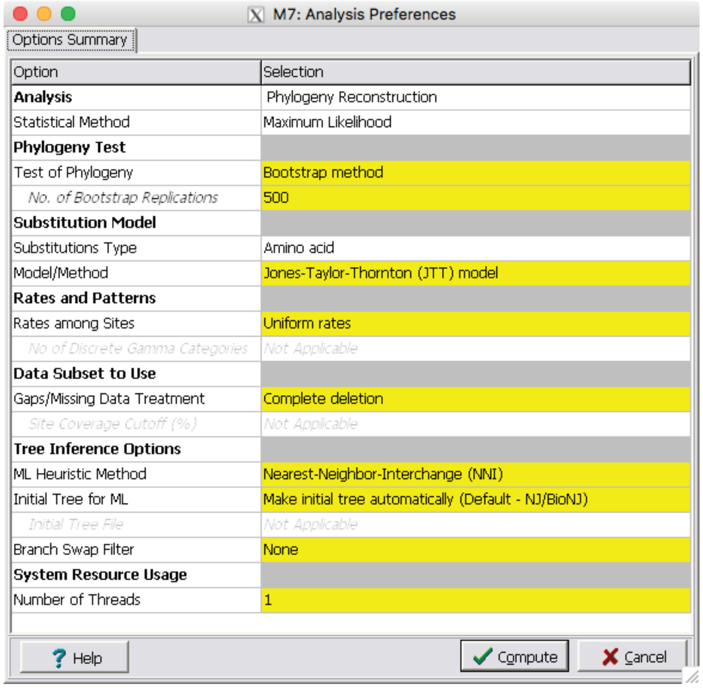

Figure S7

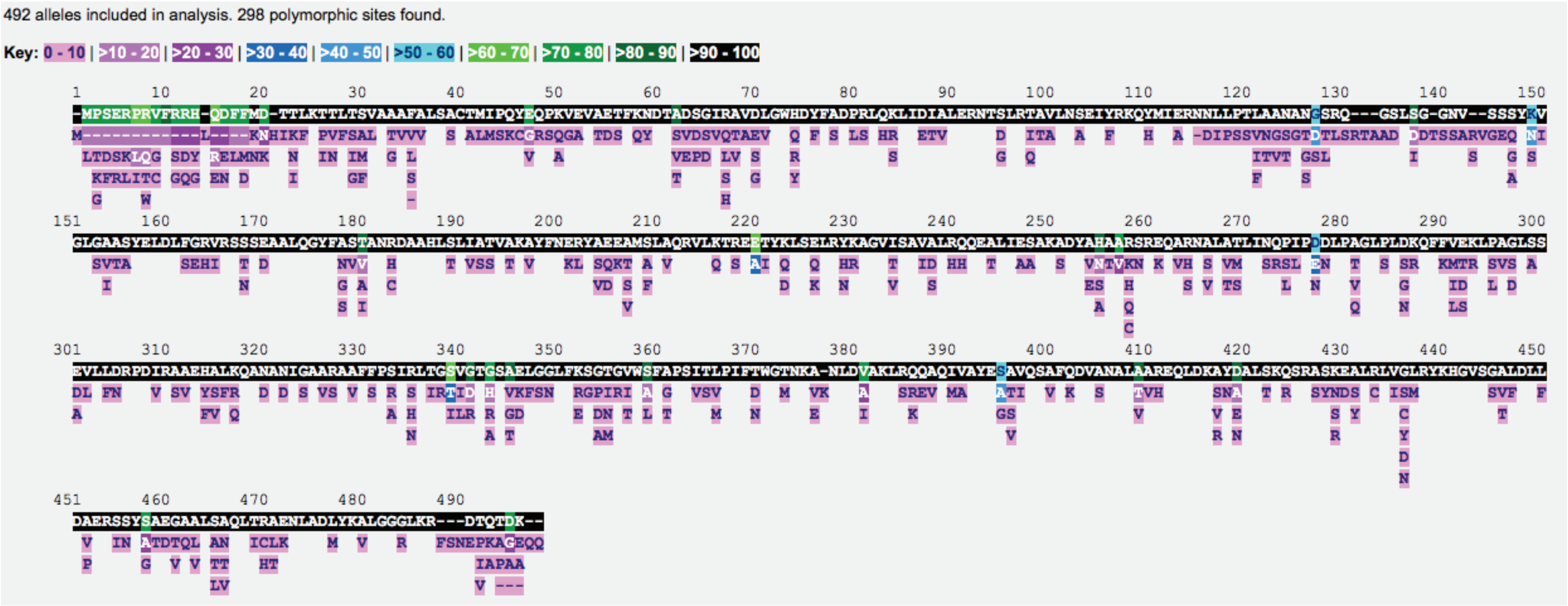
492 alleles included in analysis. 298 polymorphic sites found.
Key: 0 - 10 | >10 - 20 | >20 - 30 | >30 - 40 | >40 - 50 | >50 - 60 | >60 - 70 | >70 - 80 | >80 - 90 | >90 - 100

Figure S8

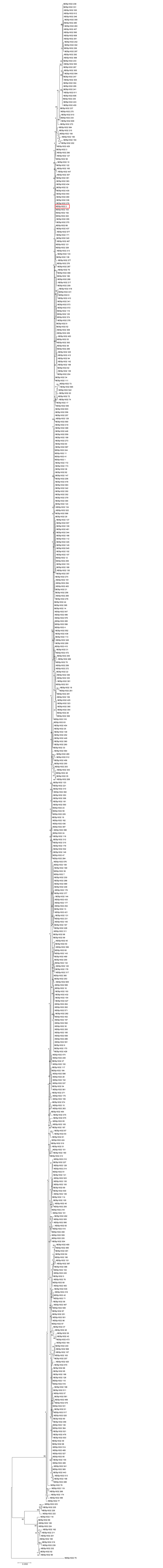

Figure S9

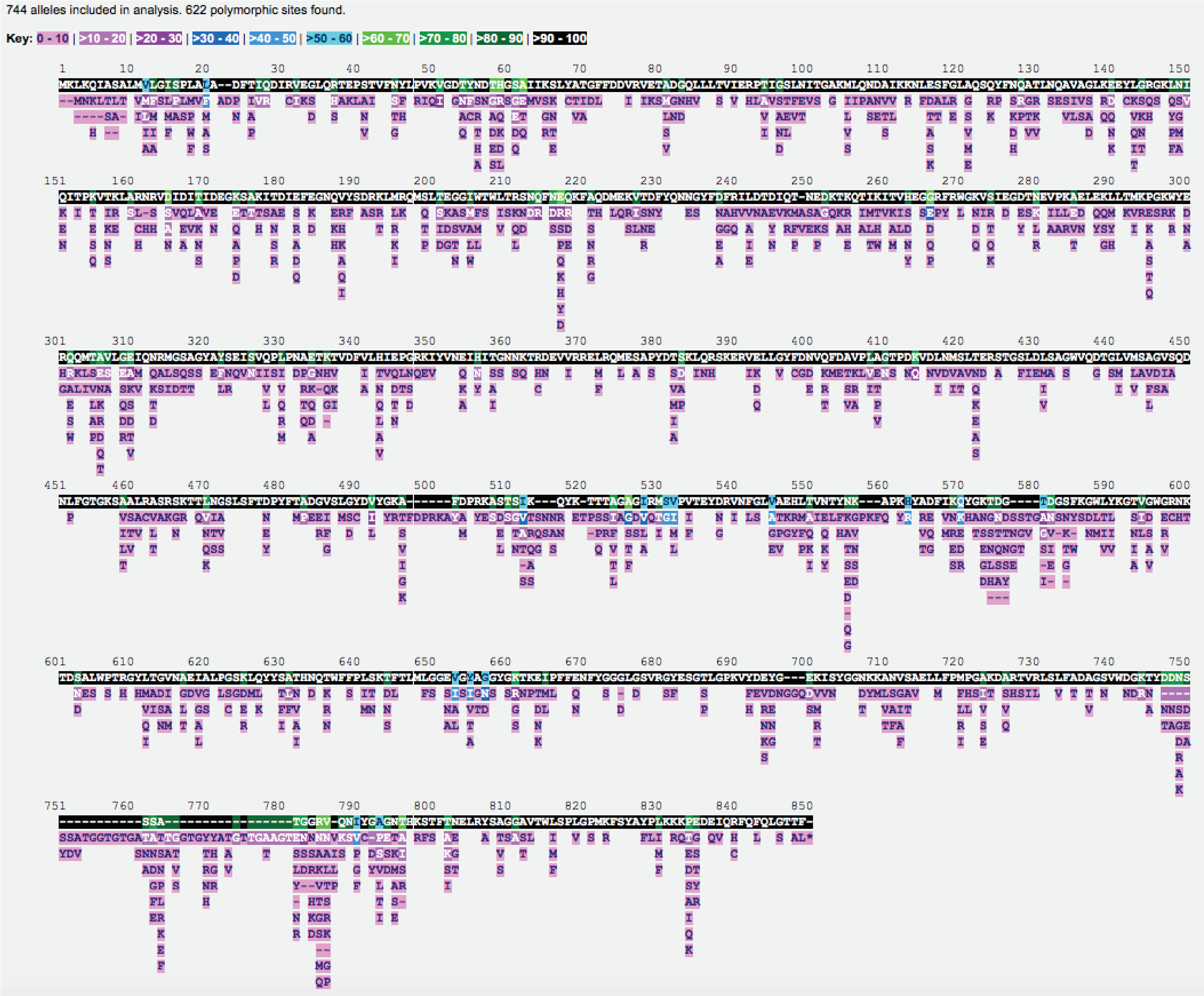
744 alleles included in analysis. 622 polymorphic sites found.
Key: 0 - 10 | >10 - 20 | >20 - 30 | >30 - 40 | >40 - 50 | >50 - 60 | >60 - 70 | >70 - 80 | >80 - 90 | >90 - 100

Figure S10

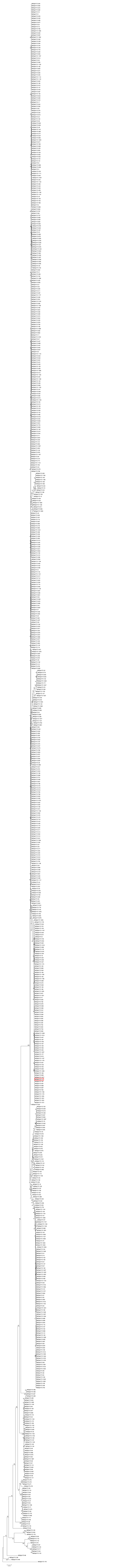

Supplement: Supplementary file 3 [file Data_Sheet_1.PDF]
